# Cerebral involvement in sitosterolemia

**DOI:** 10.1186/s12944-024-02216-8

**Published:** 2024-07-22

**Authors:** Fangjun Li, Xufang Xie, Shan Xu, Fuqing Zhou, Yaqing Yu, Xin Fang, Meihong Zhou, Min Zhu, Daojun Hong

**Affiliations:** 1 Department of Neurology, The First Affiliated Hospital, Jiangxi Medical College, Nanchang University, Yong Wai Zheng Street 17#, Nanchang, 330006 Jiangxi P.R. China; 2 Departerment of Neurology, Gaoxin Branch of The First Affiliated Hospital, Jiangxi Medical College, Nanchang University, Nanchang, Jiangxi China; 3 Department of Pathology, The First Affiliated Hospital, Jiangxi Medical College, Nanchang University, Nanchang, Jiangxi China; 4 Department of Radiology, The First Affiliated Hospital, Jiangxi Medical College, Nanchang University, Nanchang, Jiangxi China; 5 Institute of Neurology, The First Affiliated Hospital, Jiangxi Medical College, Jiangxi Academy of Clinical Medical Science, Nanchang University, Nanchang, Jiangxi China; 6 Rare Disease Center, The First Affiliated Hospital, Jiangxi Medical College, Nanchang University, Nanchang, Jiangxi China; 7 Key Laboratory of Rare Neurological Diseases of Jiangxi Provincial Health Commission, Jiangxi Medical College, Nanchang University, Nanchang, Jiangxi China

**Keywords:** Sitosterolemia, Plant sterols, *ABCG5*, Xanthoma, Neurological impairment

## Abstract

**Background:**

Sitosterolemia, an autosomal recessive condition, is characterized by impaired metabolism of plant sterols. Clinical symptoms include skin xanthoma, premature atherosclerotic disease, arthritis, and unexplained hematological abnormalities. However, there is a dearth of studies on sitosterolemia-related brain damage.

**Methods:**

This study focused on the family of two sitosterolemia patients who presented with severe hypercholesterolemia and xanthoma. Radiological examinations, biopsies, whole-exome sequencing (WES), and plant sterol tests were conducted.

**Results:**

The index patient, a 66-year-old female, initially exhibited weakness in both lower limbs and later developed urinary and fecal incontinence. Neuroimaging showed that the falx of the brain had irregular fusiform thickening. Significant tissue edema was observed around the lesions in the bilateral frontal-parietal lobes. Pathological analysis of the biopsied brain lesion revealed extensive cholesterol crystal deposition and lymphocyte infiltration in the matrix. The index patient who experienced cerebral impairment and her sister both carried two compound heterozygous variants in ATP binding cassette transporter G5 (*ABCG5*). These included the nonsense variants NM_022436: c.751 C > T (p.Q251X) in exon 6 and NM_022436: c.1336 C > T (p.R446X) in exon 10. A notable increase in plant sterol levels was observed in the younger sister of the index patient.

**Conclusion:**

This study highlights a previously unreported neurological aspect of sitosterolemia. Imaging and pathology findings suggest that cholesterol crystals may be deposited in connective tissues such as the cerebral falx and pia mater through blood circulation.

## Introduction

Sitosterolemia (OMIM 210,250), also known as phytosterolemia, is an uncommon autosomal recessive condition causing plant sterol dysmetabolism [1]. This condition arises from a biallelic loss-of-function variant in both ATP-binding cassette subfamily G member 5 (*ABCG5*) and member 8 (*ABCG8*) genes [2]. The *ABCG5* and *ABCG8* genes are positioned at the *STSL* locus on chromosome 2p21 and are approximately 400 bp apart in a head-to-head orientation [3]. These genes encode a pair of ABC half transporters that form a heterodimer (G5G8), which is transported to hepatic cells and intestinal epithelial cells, facilitating the expulsion of cholesterol and phytosterols into the biliary tract and bowel [4]. However, in sitosterolemia patients, defects in this efflux pump result in sterol accumulation in the plasma, reaching levels up to 40-fold greater than those in unaffected people [5].

Variants in *ABCG5* and *ABCG8* can cause excess plant sterols to circulate and deposit in various body systems. These sterols can penetrate the arterial endothelium, leading to the promotion of foam cell formation and the induction of atherosclerosis [6]. Additionally, they can extravasate locally from the vascular wall into connective tissues such as the skin, tendons, and fascia and occasionally into the bone membrane [7]. Mononuclear cells and macrophagic cells within these tissues take up lipid particles via specialized receptors or through phagocytosis, including aggregates of low-density lipoprotein cholesterol (LDL-C) and lipid complexes bound with antibodies, thereby forming foam cells [8]. Furthermore, excessive plant sterols circulating in the blood can be deposited in erythrocytes and platelets [9], impairing erythrocyte membrane fluidity and increasing permeability, potentially leading to erythrocyte heteromorphism and hemolysis. Plant sterols also disrupt the megakaryocyte boundary membrane system, which can result in thrombocytopenia [10, 11]. The clinical manifestations of sitosterolemia include skin xanthoma, premature atherosclerosis, arthritis, and unexplained hematological abnormalities such as hemolysis, iron deficiency anemia, macrothrombocytopenia, and splenomegaly [12, 13].

However, no reports or studies have been conducted on brain damage caused by sitosterolemia. This study describes a family with two sitosterolemia patients who exhibited severe hypercholesterolemia and xanthoma. Genetic analysis indicated that both patients had *ABCG5* variants. Neuroradiology and brain biopsy were performed in a patient with neurological deficits, and the effects of treatment were observed. These findings further broaden the phenotypic profiles of sitosterolemia patients and provide valuable insights into cerebral impairment associated with this condition.

## Materials and methods

### Subjects

The index patient was recruited from a tertiary general hospital. The subject and her relatives provided a detailed medical history. Information concerning age at symptom initiation, ailment progression, household history, and different medical manifestations was collected. The index patient underwent a number of clinical and laboratory examinations, including routine blood tests, biochemical tests, hormone level determination, cerebrospinal fluid tests, and immune-related examinations, to investigate the underlying cause of inflammation, toxicity, or metabolic abnormalities.

All tissue samples were collected after written consent was obtained from each patient in accordance with the bioethics legal guidelines of China and the Declaration of Helsinki. This research was authorized by the ethics committee of the First Affiliated Hospital of Nanchang University.

### Radiological examinations

All magnetic resonance imaging (MRI) examinations were carried out with a 3.0T MR scanner. The following sequences were used according to standard protocols: T1-weighted spin echo (T1WI), T2-weighted spin echo (T2WI), fluid-attenuated inversion recovery (FLAIR), diffusion-weighted image (DWI), apparent diffusion coefficient (ADC), susceptibility weighted imaging (SWI), and magnetic resonance angiography (MRA). Contrast-enhanced MRI was also performed at least once.

### Pathological examinations

Due to thrombocytopenia in the sitosterolemia patient with brain injury, blood smears and bone marrow cytology were obtained. Moreover, a biopsy of a subcutaneous facial mass was performed on the index patient, along with a biopsy of the Achilles tendon mass in her sister. All the samples were subjected to pathological examination.

Brain biopsies were obtained from the index patient who had cerebral impairment, and pathological examinations were conducted. The brain was fixed with 10% neutral buffered formalin, and the chosen tissues were embedded in paraffin. Paraffin-embedded blocks were then sliced into 4-µm thick sections. The brain sections were stained with hematoxylin and eosin (H&E), Klüver-Barrera (KB), periodic acid-Schiff (PAS), periodic acid silver methenamine (PASM), acid-fast bacilli, and immunohistochemical stains for a variety of antibodies concentrated on proteins related to nervous system diseases.

### Genetic sequencing

Genomic DNA was extracted from peripheral blood samples. Whole-exome sequencing (WES) was conducted by Running Gene, Inc. (Beijing, China). Targeted exon enrichment was performed using SureSelect Human All Exon V5 (Agilent Technologies). The exon-enriched DNA libraries were subjected to paired-end sequencing via the HiSeq2000 platform (Illumina). The sequenced information was aligned to the human reference genome (hg19) using the Burrows–Wheeler Aligner (BWA) [14]. Duplicate reads were eliminated using Picard tools. The Genome Analysis Toolkit (GATK) was used to identify single-nucleotide polymorphisms (SNPs) and insertions/deletions (indels) in accordance with exceptional practices. The variants were subsequently annotated with ANNOVAR. Variations with minor allele frequencies > 0.05 in public databases (such as the 1,000 Genomes Project, dbSNP, ExAC, and gnomAD) were excluded. Multiple prediction software programs, including MutationTaster, FATHMM-MKL, and CADD, were used to analyze the influence of single-nucleotide variants (SNVs), as previously described. Sanger sequencing was subsequently performed to validate the genetic variations identified via WES. The patient’s younger sister, who had xanthoma, and healthy son were included in the verification process. The target sequence was amplified via polymerase chain reaction (PCR), and Sanger sequencing was carried out using an ABI 3730 sequencer (Applied Biosystems, Foster City, CA, USA).

## Results

### Clinical features

**Fig. 1:**
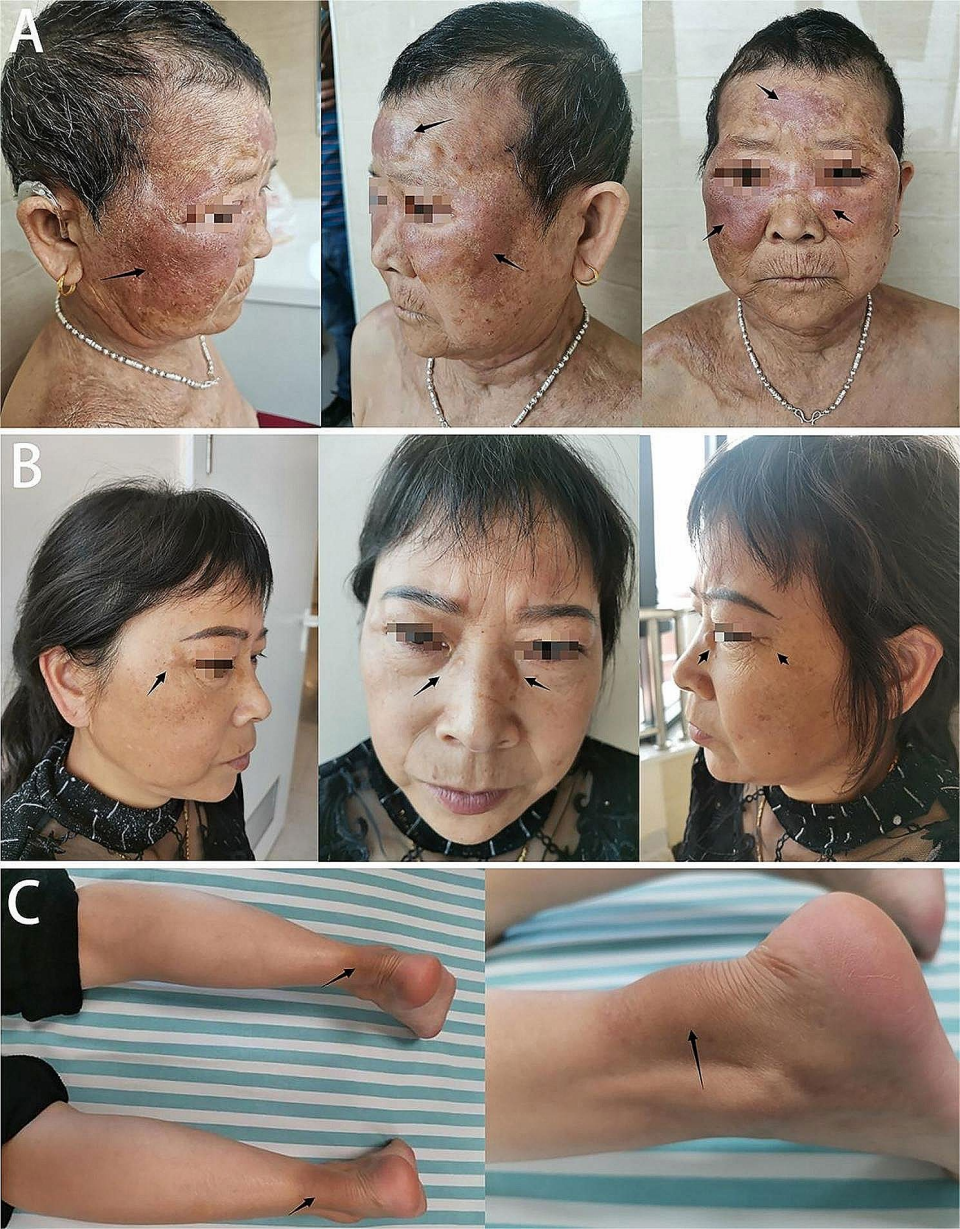
Xanthomas of sitosterolemia patients. Xanthoma can be seen around both eyelids, cheeks, and neck of the index patient (**A**). Xanthoma can be seen around the eyelids (**B**) and bilateral Achilles tendons (**C**) of the index patient’s younger sister

The index patient was a woman who experienced unexplained splenomegaly and thrombocytopenia and underwent splenectomy at the age of 37. At the age of 54, she noticed pale-yellow papules near her lower eyelids and sides of her nose, which were initially about the size of rice grains. These papules gradually increased in size; spread to both eyelids, cheeks, and neck; and formed nodules. As the disease progressed, the xanthoma extended to her neck and trunk, and the affected areas gradually expanded (Fig. 1A). At the age of 61, weakness in both lower limbs became evident, with the weakness on the left side being more severe. The patient experienced limping and often fell while crossing thresholds; however, she could still perform agricultural work without requiring assistance while walking. By the age of 66, the patient’s weakness in both lower limbs had further increased, leading to frequent falls and urinary and fecal incontinence. Her family history indicated no evidence of consanguineous marriage between her parents. Her younger sister who also exhibited xanthomas around her eyelids and bilateral Achilles tendons (Fig. 1B and C) and her healthy son were included in the study. Both the patient and her sister had a history of thrombocytopenia and hyperlipidemia.

The patient’s Mini-Mental State Examination (MMSE) score was 20/30, and her Montreal Cognitive Assessment (MoCA) score was 16/30. No signs of ptosis, nystagmus, or ophthalmoplegia were detected. Gross hearing loss affected both ears, and the patient was assisted by hearing aids. The muscle strength (Medical Research Council) of both upper limbs was grade 5; the proximal muscle strength of the left lower limb was grade 5, and the distal muscle strength was grade 4+; and the proximal muscle strength of the right lower limb was grade 5, and the distal muscle strength was grade 5-. Muscle tone was within normal limits in each upper limb, while muscle tone increased in each lower extremity. No weakness was noted in the facial, masseter, bulbar, neck, trunk, or respiratory muscles. Tendon reflexes in the extremities were normal. Babinski’s sign was suspected to be positive on the left side, whereas Babinski’s sign on the right side was negative. The patient demonstrated weakened superficial and vibration sensations below both knee joints, as well as decreased sensation of joint position. No evidence of ataxia symptoms or dysautonomia was detected. The meningeal irritation sign was negative.

### Laboratory tests

The results of the biochemical tests were as follows: total cholesterol, 10.24 mmol/L (normal 0-5.7); triglyceride, 3.64 mmol/L (normal 0-1.7); high-density cholesterol (HDL-C), 1.73 mmol/L (normal 1.29–1.55); and low-density cholesterol (LDL-C), 7.06 mmol/L (normal 0-3.62). Serum protein electrophoresis showed that the concentrations of total protein and protein fractions were as follows: albumin, 55.1% (normal, 55.8–66.1%); α1-globulin, 6.5% (normal, 2.0–4.9%); and α2-globulin, β-globulin, and γ-globulin within the normal range. Thyroid function test results were as follows: FT3, 1.93 pg/mL (normal 2.0–4.4); FT4, 0.71 ng/dL (normal 0.93–1.70); and sTSH, 9.85 µIU/mL (normal 0.27–4.2). No abnormalities in liver or kidney function, blood glucose levels, C-reactive protein (CRP) levels, sex hormone levels, erythrocyte sedimentation rate (ESR), antinuclear antibodies, or antineutrophil cytoplasmic antibodies (ANCAs) were detected. Routine blood tests revealed thrombocytopenia (platelet count: 44 × 10^9^/L, normal 125–350). Cerebrospinal fluid analysis revealed an opening pressure of 130 mmH_2_O (normal 80–180), 1 cell/µL, a protein concentration of 906 mg/L (normal 150–450), and normal sugar, biochemical, and chloride levels. Cryptococcus capsular antigen, ink staining, *Mycobacterium tuberculosis* GeneXpert and immunofixation protein electrophoresis yielded negative results.

### Instrumental tests

Chest and abdominal CT scans revealed a few chronic infection foci in the left upper lobe, little pericardial effusion, a reduced volume in the left liver lobe, the absence of the spleen, and multiple kidney stones. Nerve conduction studies revealed decreased conduction amplitude and pace of the bilateral common peroneal nerve; slightly decreased amplitude of the right median nerve and left tibial nerve; slowed sensory conduction velocity of the bilateral median, superficial ulnar, and peroneal nerves; and no elicitation of the bilateral H reflex.

**Fig. 2:**
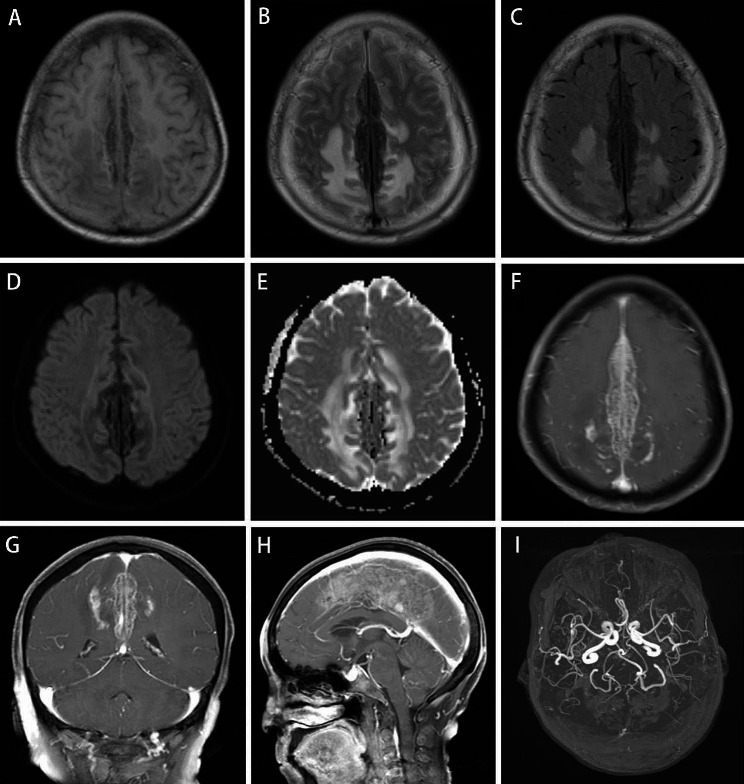
Cerebral MRI of the index patient. The falx of the brain is irregularly fusiform and thickened. Both T1WI (**A**) and T2WI (**B**) show low signals. In the FLAIR image (**C**), large hyperintensity areas can be seen in the bilateral frontal-parietal lobes around the lesion. Diffusion-weighted Iimaging, DWI (**D**). Apparent diffusion coefficient, ADC (**E**). The contrast-enhanced scan showed uneven and noticeable enhancement (**F**). Coronal view (**G**). Sagittal view (**H**). No obvious abnormalities were found on MRA of the brain (**I**)

**Fig. 3:**
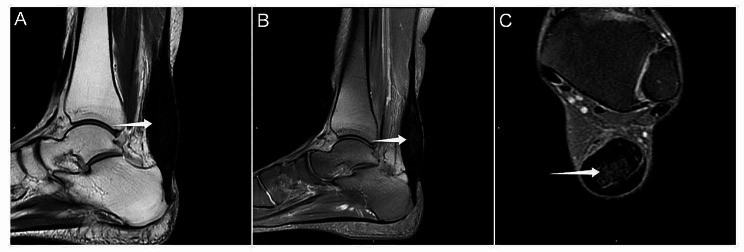
MRI of the ankle of the index patient’s sister. A Spindle-shaped enlargement of the Achilles tendon (**A**, **B**, arrow). Scattered low-signal tendon bundles can be seen inside (**C**, arrow)

On MRI, the falx of the brain exhibited irregular fusiform thickening, with dimensions of 9.4 × 3.4 × 2.1 cm, with low signals on both T1WI and T2WI. In the FLAIR image, large hyperintensity areas were observed in the bilateral frontal-parietal lobes surrounding the lesion, while an uneven and significantly enhanced signal was detected in the contrast-enhanced MR scan (Fig. 2). The soft tissue of the forehead was thickened. No abnormalities were observed on MRA of the brain. The brain MRI of the index patient’s sister showed no abnormalities, but an ankle MRI revealed spindle-shaped enlargement of the Achilles tendon with density and signal similar to that of the muscle, with scattered low-signal tendon bundles observed within it (Fig. 3).

### Pathological analysis

**Fig. 4:**
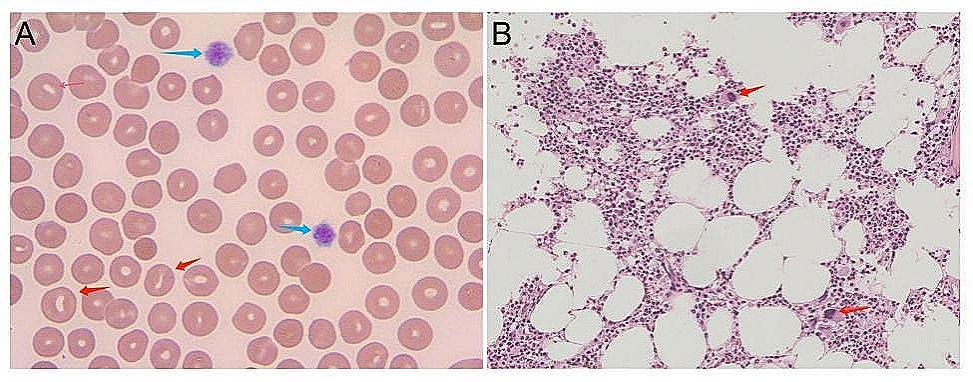
Peripheral blood smear and bone marrow cytology of the index patient. (**A**) Peripheral blood smear revealed an increase in stomatocytes (red arrow) and enlarged platelets (blue arrow). (**B**) Bone marrow cytology test showing megakaryocytes (red arrow)

A peripheral blood smear from the index patient revealed an increase in stomatocytes and enlarged platelets (Fig. 4A). Additionally, the bone marrow cytology test revealed megakaryocytosis (Fig. 4B).

**Fig. 5:**
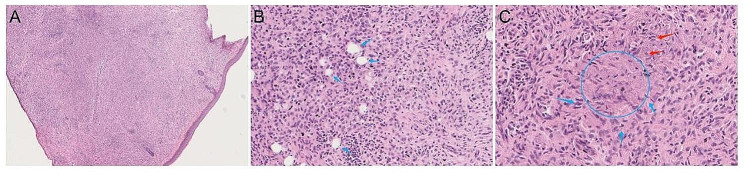
Pathology of the facial subcutaneous lump biopsy of the index patient. The lesion is located within the dermis and consists of tissue cells (**A**). Many histiocytes infiltrated, and some were engulfed with lipids, which appeared as foam-like, focal lymphocyte infiltration (**B**, blue arrow). A trend toward cholesterol crystallization (**C**, circular area) was observed in the focal area, with epithelial cells arranged in a palisade or radial pattern (**C**, blue arrow) and crystalline bodies appearing in the cytoplasm (**C**, red arrow)

**Fig. 6:**
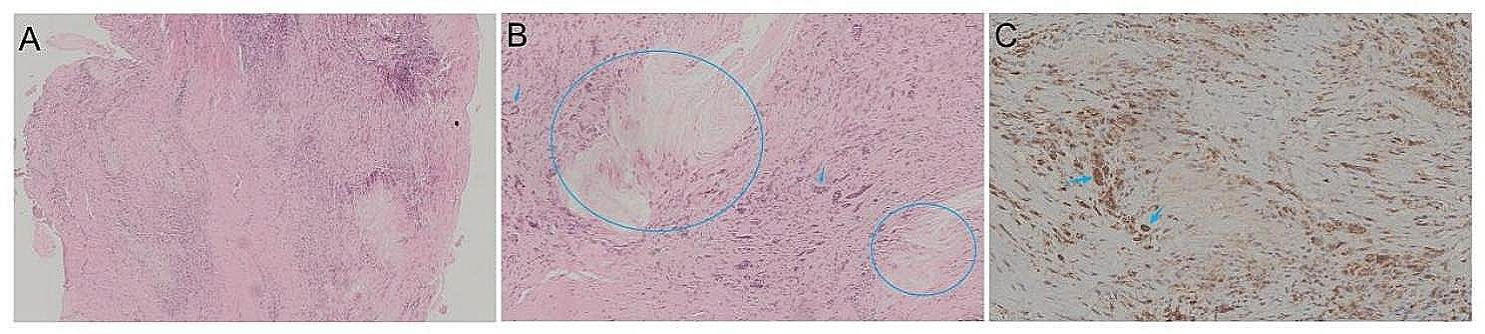
Pathology of the Achilles tendon of the index patient’s sister. The Achilles tendon is a proliferative fibrous tissue with some areas of collagen degeneration and visible cholesterol crystals (**A**). The cholesterol crystals in the Achilles tendon radiate (**B**, circular area), surrounded by many Touton cells, and the nucleus appears as a wreath (**B**, blue arrow). CD68 + Touton cells can be seen around the cholesterol crystals (**C**, blue arrow)

Pathology of the subcutaneous facial lump biopsy of the index patient revealed the following: the lesion was located within the dermis and consisted of tissue cells. The lesion was infiltrated by a large number of histiocytes, some of which were engulfed by foam-like lipids; focal lymphocytes were also observed. Moreover, there was evidence of cholesterol crystal formation in the affected area (Fig. 5). Pathology of the Achilles tendon of the index patient’s sister revealed the following characteristics: numerous foam-like cells, cholesterol crystal fissure nodules, a small number of lymphocytes, inflammatory cells, multinucleated giant cells, and Touton cells that were rich in lipid infiltration within the hyperplastic fibrous tissues. Immunohistochemistry was positive for CD68 (Fig. 6).

**Fig. 7:**
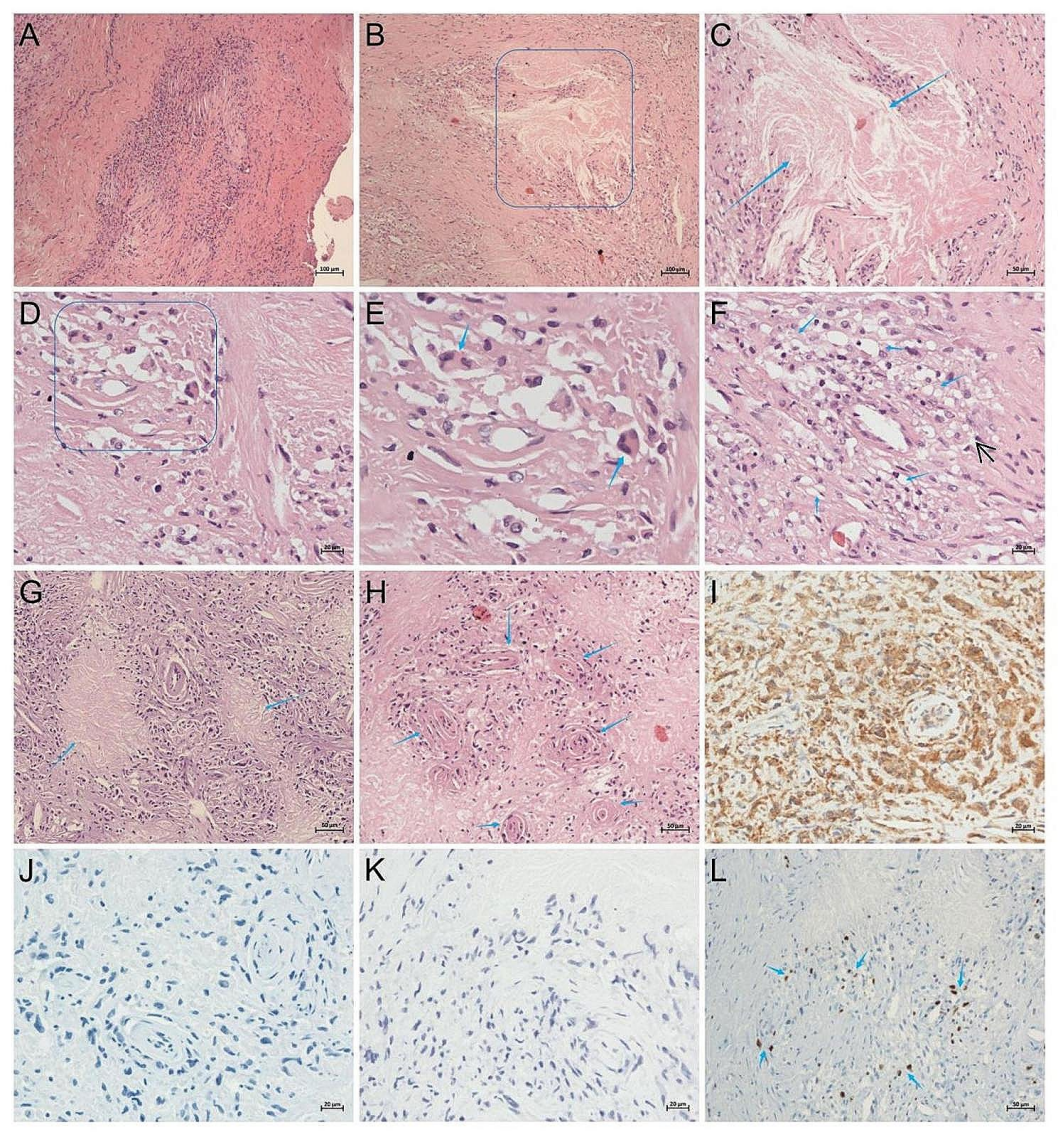
Brain lesion pathology in the index patient. Many lightly stained cells in the cytoplasm of proliferative fibrous tissue (**A**). Irregular necrotic foci, no structure in the center, and a large amount of cholesterol crystal deposition and lymphocyte infiltration in the matrix (**B**, rectangular area), Enlarged image of the enclosed area in B (**C**, blue arrow). Multinucleated giant cells forming a granulomatous structure (**D**, rectangular area). Enlarged image of the enclosed area in D (**E**), with Touton cells full of lipids (blue arrow). Foam cell (**F**, blue arrow). Lymphocyte granuloma infiltration (**G**, blue arrow). Vascular wall thickening with hyaline degeneration (**H**, blue arrow). CD68-positive staining (**I**). S-100-negative staining (**J**). CD1a-negative staining (**K**). Ki-67 5% positive staining (**L**, blue arrow)

Biopsies were performed on the index patient’s intracranial lesions. The lesion in the left parietal lobe, observed after craniotomy, appeared as a grayish-yellow bulge with a soft texture, unclear boundaries, and visible calcification. The final pathological analysis of the brain lesion biopsy showed a substantial number of lightly stained cells within the cytoplasm of proliferative fibrous tissue. Irregular necrotic foci were present, lacking structure at the center. Multinucleated giant cells and typical Touton cells were observed in the surrounding area and formed a granulomatous structure. Moreover, significant cholesterol crystal deposition and lymphocyte infiltration were present in the matrix, as well as vascular wall thickening and hyaline degeneration. Immunohistochemistry results were negative for GFAP, EMA, PR, E-cd, S-100, CD1a, and CD34 and positive for CD68, INI-1 (5%), and Ki-67. The PAS, PASM, and acid-fast special stains were all negative (Fig. 7).

### Genetic analysis

**Fig. 8:**
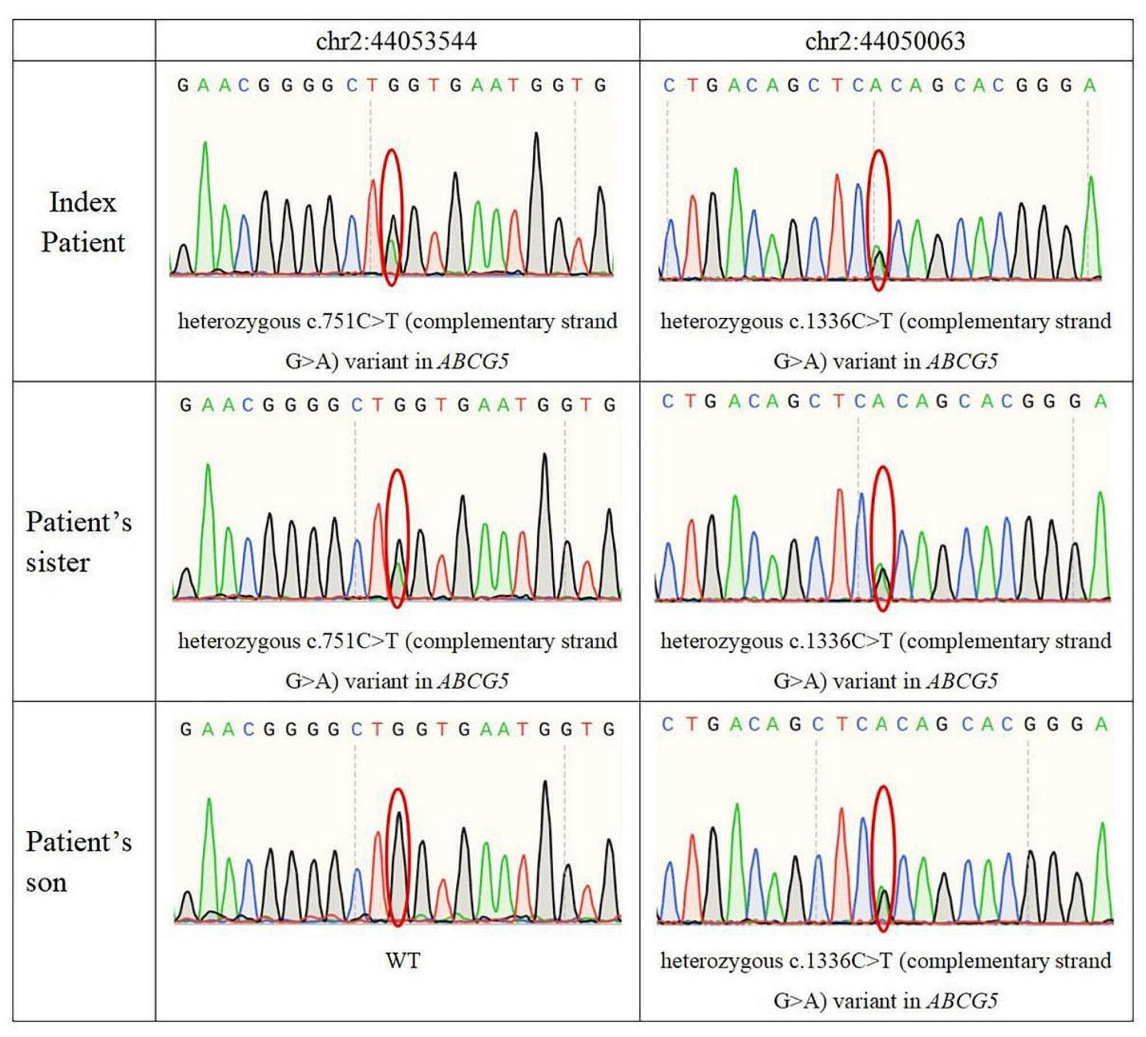
Whole-exome sequencing results. The nonsense variant NM_022436: c.751 C > T (p.Q251X) in exon 6 and NM_022436: c.1336 C > T (p.R446X) in exon 10 were detected in the index patient and the patient’s sister. The patient’s healthy son had one nonsense variant, NM_022436: c.1336 C > T (p.R446X) in exon 10

Using hierarchical filtering of next-generation sequencing (NGS) data, *ABCG5* gene variants were identified. The WES results were confirmed via Sanger sequencing. Compound heterozygous variants were found in the *ABCG5* gene within this family. Both the sitosterolemia patient with cerebral impairment and her younger sister exhibited a combination of two heterozygous variants identified in *ABCG5*. These variants included the previously reported nonsense variants NM_022436: c.751 C > T (p.Q251X) in exon 6 and NM_022436: c.1336 C > T (p.R446X) in exon 10. Additionally, the healthy son of the patient harbored one nonsense variant, NM_022436: c.1336 C > T (p.R446X), in exon 10 (Fig. 8). Several prediction software programs, including MutationTaster, FATHMM-MKL, and CADD, were used to analyze the impact of SNVs, and the results of these analyses are shown in Table 1. According to the ACMG classification and publication data, these variants were classified as pathogenic.

**Table 1:** Predictive software programs analyze the impact of SNVs

Variant	Mutation taster	FATHMM-MKL	CADD
c.751 C > T p.Q251* (chr2-44053544)	Disease causing (prob: 1)	Disease causing (Non-coding score: 0.99521 Coding score: 0.99493)	Disease causing (PHRED: 45)
c.1336 C > T p.R446* (chr2-44050063)	Disease causing (prob: 1)	Disease causing (Non-coding score: 0.98429 Coding score: 0.94346)	Disease causing (PHRED: 40)

### Follow-up

**Fig. 9:**
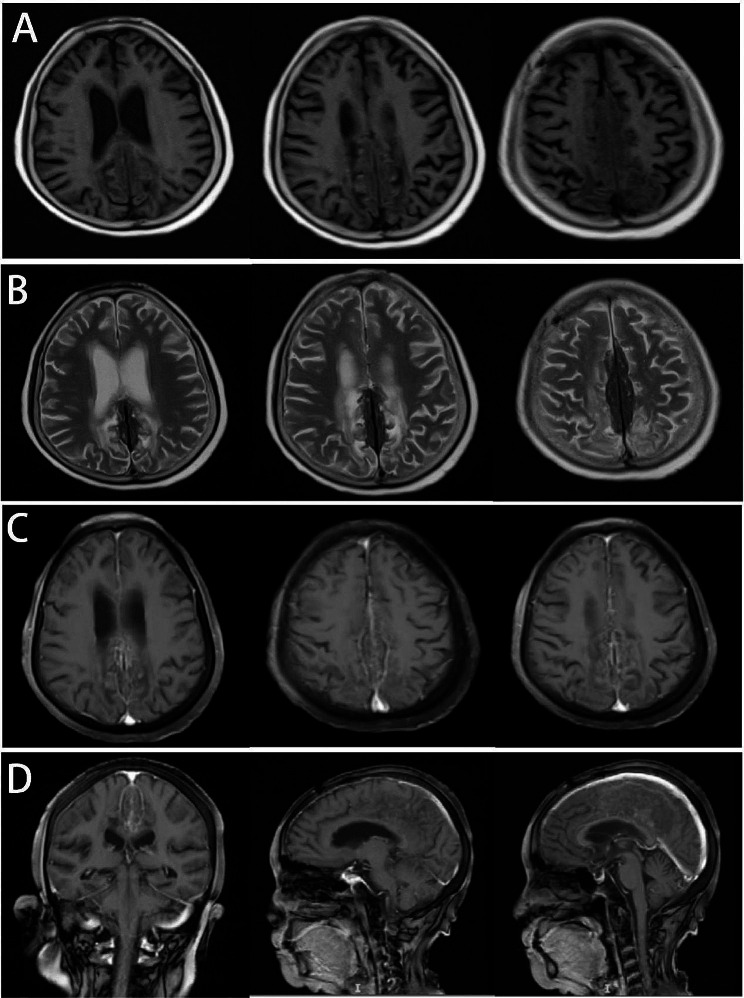
Cerebral brain MRI of the index patient one year after treatment. Both T1WI (**A**) and T2WI (**B**) show that the irregular fusiform thickening of the falx cerebri is smaller than before, and the patchy high-signal edema area around the lesion is significantly reduced (**B**). Compared with the enhanced scan before treatment, the enhancement was not apparent (**C**, **D**)

The index patient received ezetimibe (10 mg, once daily) and cholestyramine (4 mg, three times daily) after diagnosis, and was initiated on a low-cholesterol and low-phytosterol diet. The index patient was followed up one year later. Her lower limb weakness improved slightly. Blood lipid analyses revealed a total cholesterol level of 5.93 mmol/L (normal 0-5.7), a triglyceride level of 2.48 mmol/L (normal 0-1.7), a high-density cholesterol (HDL-C) level of 1.9 mmol/L (normal value 1.29–1.55), and a low-density cholesterol (LDL-C) level of 3.02 mmol/L (normal value 0-3.62). Brain MRI revealed a reduced range of irregular fusiform thickening in the cerebral falx, a significant reduction in the patchy high-signal edema area around the lesion, and no significant enhancement on the enhanced scan (Fig. 9). Phytosterols were subsequently analyzed to obtain comprehensive results. However, due to the limited mobility of the index patient, her blood sample could not be collected for examination. Unfortunately, she passed away from COVID-19 in 2023 at the age of 68. The results for the younger sister of the index patient were as follows: cholestanol, 51.22 µmol/L (0.01–10.00); desmosterol, 11.23 µmol/L (0.30–5.00); campesterol, 749.15 µmol/L (0.01–10.00); stigmasterol, 53.46 µmol/L (0.10–8.50); sitosterol, 523.60 µmol/L (1.00–15.00); squalene, 0.76 µmol/L (0.30–4.00); and lathosterol, 3.18 µmol/L (0.01–12.50).

## Discussion

This study investigated a family with compound heterozygous variants in the *ABCG5* gene. The presence of xanthomas on the skin and tendons, hypercholesterolemia, and significantly elevated phytosterol levels in the patients, combined with the presence of *ABCG5* gene variants, met the diagnostic criteria for sitosterolemia. In particular, the index patient developed rare neurological damage, which was confirmed by neuroimaging and brain biopsy. The clinical symptoms and neuroimaging findings improved following treatment with ezetimibe and cholestyramine.

Sitosterolemia is an uncommon genetic lipid abnormality, with an estimated incidence ranging from 1 in 360,000–2.6 million [15]. Previous studies have indicated that most Asian sitosterolemia patients, particularly Chinese or Japanese individuals, harbor the *ABCG5* variant, whereas Caucasians harbor the *ABCG8* variant [10]. Within the Chinese population with sitosterolemia, nearly half of the patients with *ABCG5* variants exhibit the R446X variant, which is the most common variant in this population. In this particular case, the index patient carried the heterozygous nonsense variants Q251X and R446X, resulting in the truncation of the coding protein and a severe impact on its function. The Q251X variant is relatively rare, but multiple patients have been documented in the literature since 2017. Variations in the *ABCG5* gene reduce plant sterol excretion into the bile and bowel, leading to its accumulation in plasma and tissues. An alternative explanation is that given the abundant expression of G5G8 in the intestinal epithelium, *ABCG5* variants also affect the frequent pathway for the absorption of cholesterol and phytosterol, which involves Neiman-Pick C1-Like-1 (NPC1L1) [16], causing increased transport of sterols from the intestinal lumen into enterocytes. Consequently, ezetimibe and bile acid sequestration resins have become standard treatments, both of which effectively reduce sitosterol levels (∼ 20% with ezetimibe and ∼ 30% with resins) and lower LDL-C in patients with sitosterolemia [2].

Lipid metabolism disorders, including xanthoma, arteriosclerosis, arthritis, hepatomegaly and splenomegaly, stomatocytosis, megathrombocytosis, and thrombopenia, share similar clinical manifestations [12, 13]. Sitosterolemia is frequently confused with homozygous familial hypercholesterolaemia (HoFH) due to elevated plasma cholesterol values and the presence of xanthomas; more rarely it can be confused with the heterozygous FH (HeFH) in adults [2]. In sitosterolemia, dietary treatment results in an important reduction in cholesterol values, however, the effect of dietary intervention on familial hypercholesterolaemia (FH) is small (10–12% from baseline); similarly, the use of drugs such as cholestyramine and/or ezetimibe leads to normalisation of plasma total cholesterol and LDL cholesterol values in sitosterolemia, whereas FH usually requires combined treatment with statins and ezetimibe or proprotein convertase subtilisin/kexin type 9 (PCSK9) inhibitors. The etiology of FH involves various genetic mutations. Familial defects in apolipoprotein B-100 are caused by variants in *APOB*, familial hypercholesterolemia is caused by variants in the LDL receptor (*LDLR*), and non-FH/non-FDB hypercholesterolemia is caused by variants in *PCSK9.* Furthermore, lipid storage disorders other than cerebrotendinous xanthomatosis (CTX) rarely result in neurological damage. CTX arises from mutations in the sterol 27 hydroxylase (*CYP27A*) gene and leads to progressive neurological dysfunction [8]. Consequently, gene sequencing is essential for accurately distinguishing between lipid storage diseases.

Previously, it was believed that patients with sitosterolemia did not experience neurological impairment [17]. To our knowledge, there have been no reports of intracranial involvement in sitosterolemia. However, the index patient presented with irregular fusiform thickening of the cerebral falx. Additionally, there were large areas with high FLAIR signals, indicating significant tissue edema in the bilateral frontal lobes surrounding the lesion. Enhanced scans revealed enhancement of the lesion, suggesting that the blood‒brain barrier may be disrupted or that the blood supply may be increased. The lesion grew along the falx cerebri and pia mater space, invading and proliferating, resulting in edema and a space-occupying effect. It is speculated that intracranial lesions may be associated with the deposition of cholesterol crystals in connective tissues, such as the cerebral falx and pia mater, through the bloodstream. Plant sterols have been shown to penetrate the arterial endothelium, promote foam cell formation, induce atherosclerosis, and trigger inflammatory reactions, ultimately leading to the formation of lipid granulomas. Neuroimaging revealed enhanced focus and space-occupying effects. One year after treatment, a significant reduction in the lesions was observed on neuroimaging.

Neuropathology analysis revealed numerous microstained cells, proliferative fibrous tissue, irregular necrotic lesions, unstructured multinucleated giant cells, granulomatous structures, abundant cholesterol crystal deposits, and lymphocyte infiltration in the thickened vascular wall with hyaline degeneration. GFAP-negative cells can exclude glial cell-derived diseases. EMA-negative, PR-negative, and E-cd-negative cell staining can exclude meningioma. The CD34-negative, INI-1 (partial positive), and Ki-67 (5% positive) staining can exclude angiosarcomatosis. PAS-negative, PASM-negative, and acid-fast-negative stains can exclude fungal and tuberculosis infections.

CD68-positive staining suggested the presence of numerous histiocytes in the perivascular and interstitial tissues, indicating histocyte-related diseases. However, CD1a-negative and S100-negative cells can exclude indeterminate cell histiocytosis (ICH) and Langerhans cell histiocytosis (LCH) but they cannot be used to distinguish necrobiotic xanthogranuloma (NXG) and juvenile xanthogranuloma (JXG). NXG characterized as a non-Langerhans histiocytosis, is a progressive adverse disorder that exhibits paraproteinaemia, multiple organ engagement, and heightened susceptibility to hematological and lymphoproliferative malignancies [18]. There has been a previous report of NXG with an intracranial lesion [18]. Histological examination of the cerebral lesion revealed sheets of uniform slim spindle cells organized in a storiform pattern within a collagenous matrix. Lymphocytes, plasma cells, and eosinophils were observed and were predominantly located around the blood vessels. Additionally, sheets of CD68-positive foam cells were present in many places. Lymphoid follicles were observed, and multinucleated giant cells occasionally appeared [18]. The index patient in the study presented with a facial lump with histology indicative of a histiocytosis lesion and a brain biopsy revealing a granulomatous structure, which was easily mistaken for NXG. Relying on these pathological findings, the patient was initially diagnosed with NXG before the genetic results were obtained and was given immunotherapy (gamma globulin, cyclophosphamide, and corticosteroids), but no significant improvement was observed after one month. In terms of clinical manifestations, both NXG and sitosterolemia are characterized by xanthomas, increased blood lipids, and thrombocytopenia; in terms of pathological characteristics, both diseases are also characterized by the deposition and destruction of lipid cells in tissue cells. However, serum protein electrophoresis and bone marrow cytology results from the index patient did not support this diagnosis, and immunotherapy yielded no discernible effect. Subsequent genetic analysis provided the correct diagnostic direction.

## Strengths and limitations of the study

This study is the first to investigate the relationship between sitosterolemia and brain damage. Several limitations should also be noted in this study. First, the data collection was not complete, specifically the inability to obtain blood samples from the index patient for phytosterol level measurements. Second, the study focused on a family with sitosterolemia carrying *ABCG5* variants, and brain involvement was complicated by the coexistence of different pathological conditions; therefore, larger clinical studies involving additional patients are necessary to validate this association. However, given the rarity of this disorder, enrolling additional patients poses a challenge. Third, conducting functional experiments, either in vitro or in vivo, is crucial for understanding how *ABCG5* variants impact protein function related to brain impairment in patients with sitosterolemia. These aspects will be explored in future research. Finally, due to the absence of prior studies on sitosterolemia patients with brain impairment, comparisons with previous research were not feasible.

## Conclusion

In summary, the primary manifestations of sitosterolemia include skin xanthoma, premature atherosclerotic disease, arthritis, and unexplained hematological abnormalities. In the present study, the index patient showed symptoms of neurological deficits. Neuroimaging showed that the falx of the brain exhibited irregular fusiform thickening. Significant tissue edema was observed around the lesions in the bilateral frontal-parietal lobes. Pathological analysis of the biopsied brain lesion revealed high cholesterol crystal deposition and lymphocyte infiltration in the matrix. The patient’s neurological impairment improved after one year of treatment with ezetimibe and cholestyramine. These findings seems to support a cerebral involvement of sitosterolemia and opens up new perspectives for the study and investigation of this rare disease.

## Data Availability

No datasets were generated or analysed during the current study.
